# P02.104. Nonlinear parameters of heart rate variability (HRV) – suitable measures to observe physiological outcome during a peat bath in rehabilitation

**DOI:** 10.1186/1472-6882-12-S1-P160

**Published:** 2012-06-12

**Authors:** H Janik, C Mau, K Kraft

**Affiliations:** 1 Univ. of Rostock, Rostock, Germany; 2 Rehab hospital „Moorbad" Bad Doberan KG, Bad Doberan, Germany

## Purpose

Hot peat baths (40 - 45 °C) are used in rehabilitation medicine to treat pain, increase perfusion and relax muscles. Because of substantial heat stress (core body temperature increased by 1 - 1.5 °C within 30 min) patients´ linear HRV parameters are influenced due to sympathovagal activity. The aim of the observational study was to investigate reactions of the cardiac autonomic nervous system detected by means of nonlinear parameters of HRV during a peat baths procedure.

## Methods

Cardiovascular healthy non-smoking patients (45.2 ± 8.2 years, 10 male, 19 female) with herniated vertebral discs received a peat bath (40.5 °C) in a rehab hospital. Each bathing procedure consisted of 20 minute recumbent rest (R), 20 minute peat bath (B), and a second 20 minute recumbent rest (S). RR-intervals were determined by a 24 h-ECG-system (Ela medical®, SORIN GROUP, France). For calculations of the HRV-parameters the last 6.5 min of R, and consecutive subdivisions B1, B2, B3 and S1, S2, S3 (each 6.5 min) were used. Kubios HRV 2.0 software were applied (Kuopio, Finland).

## Results

In the detrended fluctuation analysis (DFA) alpha1 increased from R to B2 continuously (p < 0.05) and sustained (S2, S3, p < 0.01). The sample entropy values (SampleEn) decreased from R to S1 (p < 0.001) and returned slowly (S2, p < 0.05). The Shannon entropy (ShanEn) increased from R to B3, S1 (p < 0.01) and returned rapidly (S2, p > 0.05). In comparison also to linear parameters, correlations provided between alpha1 and LF%, HF% (r = 0.74, p < 0.05), SampleEn and ShanEn (r = 0.86, p < 0.05), but not for heart rate.

## Conclusion

Nonlinear parameters of HRV may reflect reactions of the cardiac autonomic nervous system during a peat bath. Our results support their usage as predictors in clinical studies to improve performance.

